# Heart failure in patients with metabolic syndrome X

**DOI:** 10.15190/d.2023.1

**Published:** 2023-03-03

**Authors:** Precious-Peculiar Olatunbosun, Ghalib Nashaat El Hunjul, Apurva Patel, Rabab Hunaid Abbas, Shefali Mody, Ahmad Masalha, Shivani Mehta, Shaista Rizwan, Aayushi Pareek, Suhani Jain, Silmy Bakzer Cherat Parambat

**Affiliations:** ^1^University of Ilorin, College of Health Sciences, Ilorin, Nigeria; ^2^Department of Internal Medicine, Soba University Hopsital, Khartoum, Sudan; ^3^Medical College Baroda, Vadodara, India; ^4^Tbilisi State Medical University, Tbilisi, Georgia; ^5^Lokmanya Tilak Municipal Medical College, Mumbai, India; ^6^Ross University School of Medicine, Two Mile Hill, St. Michael, Barbados; ^7^Xavier University School of Medicine, Aruba; ^8^University of Perpetual Help System, Dalta, Philippines; ^9^Grant Government Medical College and Sir JJ Group of Hospitals, Mumbai, India

**Keywords:** Metabolic syndrome, insulin resistance, obesity paradox, heart failure, obesity.

## Abstract

Metabolic syndrome X has been known to be a risk factor for the development of cardiovascular dysfunction. Insulin resistance, diabetes mellitus and serum lipid abnormalities, which are all seen in metabolic syndrome X, have been found to negatively impact heart function, leading to heart failure in particular. Heart failure is a condition resulting when the heart is unable to perform its function of providing sufficient blood flow to meet the body’s requirements. The treatment of heart failure in metabolic syndrome X varies based on the various components of metabolic syndrome X, which include obesity, hyperglycemia, hypertension and dyslipidemia. Obesity is regarded as one of the derangements seen in patients with metabolic syndrome X. It is a significant risk factor in the development of cardiovascular disease, which may eventually lead to heart failure. However, the obesity paradox suggests that obesity provides a higher chance of survival in patients with metabolic syndrome and heart failure. This review article focuses on the pathophysiology of heart failure in patients who already have metabolic syndrome X, as well as the therapeutic management complexity of the two conditions taking into consideration the protective role provided by obesity.

## SUMMARY

*1. Introduction*

*2. Prevalence and incidence*

*3. Morbidity and mortality*

*4. Association of heart failure and metabolic syndrome X*

*5. Pathogenesis of heart failure in metabolic syndrome X patients*

*6. Diagnosis*

*7.* *Heart failure’s treatment in metabolic syndrome X*

*8. Prognosis*

*9. Conclusion*

## 1. Introduction

Metabolic syndrome, also known as insulin resistance syndrome or metabolic syndrome X, is a multifactorial syndrome affecting men more than women, and is mainly influenced by hormonal, genetic, and lifestyle factors^1^. It is a steadily increasing metabolic abnormality, affecting 34% of the general population and may lead to glucose abnormality and significant cardiovascular complications such as high blood pressure and dyslipidemia that may increase the risk of heart failure, affecting both therapeutics and follow-up management plan^2,3^. Heart failure (HF) is a chronic, progressive condition in which the heart muscle is incapable of pumping enough blood to meet the body’s needs for blood and oxygen. Heart failure is a major health concern that has been a subject of interest since it was designated as an emerging epidemic in 1997^4,5^. Since then, the total number of cases has evolved and become a major cause of mortality, hospitalization, and health care expenses among patients older than 65 years^6^. The presence of heart failure along with other comorbidities significantly affects the long-term morbidity and mortality of symptomatic and asymptomatic patients.

Moreover, metabolic syndrome may cause obesity, which is an established risk of heart failure, but recently it has been published that such a relationship may result in a better prognosis. This is called the *obesity paradox. *Obesity has been found to increase short and medium-term prognosis in association with the cardiorespiratory capacity of the patients. Several therapeutic effects including better physical activity and diet resulted in better outcomes in heart failure patients^7^. To date, the exact mechanism of metabolic syndrome and its association with heart failure and the obesity paradox are still not well understood. In this review article, we aim to highlight current knowledge regarding the impact of metabolic syndrome on heart failure development in relation to the obesity paradox.

## 2. Prevalence and incidence

The prevalence of metabolic syndrome has significantly increased over the years. According to the national health and nutrition examination Survey (NHANES), the prevalence has increased from 36.3% in 2011-2012 to 38.2% in 2017-2018 in the USA^8^. Furthermore, older studies have supported this finding as well, the prevalence was 23.1 % which increased to 27 % in 1999-2000^9^. As per the national cholesterol education program (NCEP) criteria, metabolic syndrome is so common in the US that 43.5% of the total over 50 years of age population have it^10^.

The increasing prevalence of metabolic syndrome also leads to a growing prevalence of the global pandemic, heart failure, which affects around 26 million people in the whole world^11^. Metabolic syndrome is a risk factor for other cardiovascular conditions as well, which contributes to the increase in their prevalence.

According to a systematic review and meta-analysis that were done in Iran, metabolic syndrome was present in 34.2% of a total of 44,735 cardiovascular patients^12^. Moreover, a study of heart transplant patients showed that 42.3% patients had metabolic syndrome along with ischemic heart disease before the transplant^13^. One study suggested that the prevalence of metabolic syndrome was 49.5% among 200 coronary artery disease (CAD) patients who had undergone coronary angiography^14^. The population with an age of more than 50 years in NHANES was divided into two groups depending on the presence of the metabolic syndrome and the group with metabolic syndrome showed the highest prevalence of coronary heart disease (CHD)^15^.

Similar to other cardiovascular diseases, the prevalence of the metabolic syndrome is also high in heart failure patients. Various studies have shown similar findings. One single-center study showed that out of 865 patients with indigent heart failure, 40% had metabolic syndrome^16^. A Japanese study with 3603 patients enrolled with chronic heart failure showed the presence of metabolic syndrome in 49 % of males and 19 % of females, which was higher than double than the general population. Another study with 4762 HF patients had metabolic syndrome prevalence of 41.3%^17^.

## 3. Morbidity and mortality

The presence of comorbidities significantly affects long-term morbidity and mortality of symptomatic and asymptomatic heart failure patients^18^. Population studies have shown that metabolic syndrome increases the risk of developing heart failure and this effect is mediated by insulin resistance. However, obesity is a key component in metabolic syndrome and a customary partner of insulin resistance because it is protective in patients with established heart failure, although insulin resistance confers an increased risk of dying from heart failure. This phenomenon is called the "obesity paradox" accounting for the complexity of the heart failure-metabolic syndrome relationship^6^. Metabolic syndrome has a prevalence in the general population of 27% and is associated with increased mortality. In a cohort of hospitalized patients with heart failure, the prevalence of metabolic syndrome was 68%, exceeding that of the general population. However, unlike the general population, metabolic syndrome is associated with lower mortality in heart failure patients, establishing that mortality was lower in those with metabolic syndrome (44%) compared to those without (58%, unadjusted HR 0.67). Finally, in a fully adjusted model, there was a significantly lower risk of mortality in those with metabolic syndrome (adjusted, HR 0.73)^19^. Therefore, physicians treating patients with heart failure need to address the present metabolic syndrome epidemic.

## 4. Association of heart failure and metabolic syndrome X

For more than 50 years, there has been a correlation between obesity and atherosclerotic heart disease. The obesity pandemic has made this aspect of the population's risk for cardiovascular disease more important and evident^20^. Though generally known, the link between obesity and congestive heart failure is still not fully understood. Obstructive sleep apnea and systemic and pulmonary hypertension were thought to be the primary causes of this illness, which was formerly known as "obesity cardiomyopathy”^21,22^.

Numerous studies show that diabetes mellitus and insulin resistance are not the only risk factors for heart failure. They are also associated with a more severe form of left ventricular dysfunction and higher mortality rates than people without diabetes mellitus or insulin resistance or heart failure^23^. In individuals with heart failure, insulin resistance is very common (up to 60%), and there is a complex pathophysiological interplay between these two disorders since insulin resistance may be both a cause and an effect of heart failure. Similar to heart failure patients, diabetes mellitus is widespread among them, with prevalence rates in hospitalized patients ranging from 10% to 40%^24^.Several functional, metabolic, and anatomical changes brought on by insulin resistance and diabetes mellitus ultimately lead to heart failure.

Hypertension affects 6 to 10 percent of heart failure patients. According to the model put forth by Vasan and Levy, hypertension is not known to affect LV function in the early stages. A chronic increase in pressure load caused by mechanical stress leads to a rise in the level of cytokines, neurohormones and growth factors which progress to the development of concentric LV hypertrophy, leading to symptomatic heart failure with normal or abnormal LV function^25^. The lipid abnormalities mentioned in the definition of metabolic syndrome include low plasma levels of high-density lipoprotein (HDL) and high plasma levels of triglycerides. Velagaleti et al. found that low HDL plasma levels were linked to a 40% higher risk of developing heart failure over a 26-year follow-up in a population analysis from the Framingham cohort. Consequently, lipid buildup in the heart leads to heart failure^26^.

**Figure 1:**
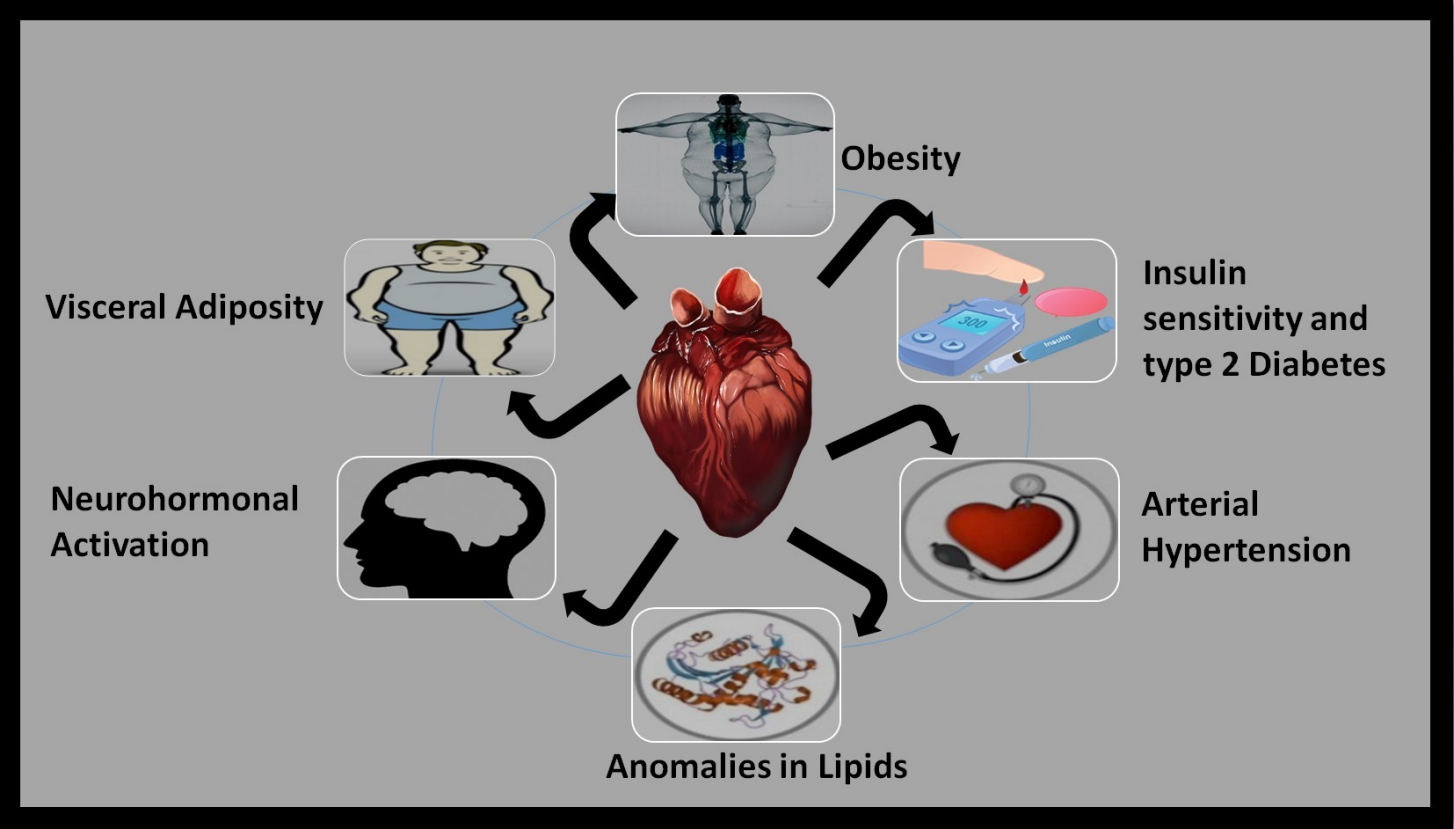
Association between heart failure and metabolic syndrome X

According to Jackson's heart research, visceral obesity is linked to an increased risk of developing heart failure in the black population^27^. The homozygous TT genotype of the aminopeptidase M1 (apM1) gene, which was identified in abundance in adipose tissue, was linked to a greater risk of metabolic syndrome. Adiponectin, a protein that resembles collagen, was also encoded by the apM1 gene^28^. Persistent activation of the sympathetic nervous system and renin-angiotensin-aldosterone system were the processes involved in metabolic syndrome^29^. Inability to adapt the results of these processes in cardiomyocyte hypertrophy, which, if the problem persists, could cause remodeling and finally result in heart failure^30^. The association between heart failure and metabolic syndrome has been illustrated in Figure 1.

## 5. Pathogenesis of heart failure in metabolic syndrome X patients

Adipose tissue is classified according to its location: subcutaneous or visceral. However, it was found that visceral adiposity was independently associated with the incidence of hospitalized patients with heart failure with preserved ejection fraction (HFpEF). Obesity and in particular central adiposity directly correlates with increased left ventricular stiffness, which contributes to the diastolic dysfunction in HFpEF. Visceral adipose tissue (VAT), which is stored in the abdominal cavity and accounts for 20% of adipose tissue, is pro-inflammatory and increases cardiovascular risk by promoting metabolic diseases such as diabetes mellitus, dyslipidemia, and hypertension. Obesity-related biomarkers such as adipokines also have prognostic value in heart failure risk but it is unknown if these biomarkers better predict heart failure than anthropometrics and CT-measured adiposity^31^.

Thus, chronic inflammation also plays an important role in heart failure associated with metabolic syndrome. Serum IL-6 and TNF-α levels are high in patients with metabolic syndrome and elevated proinflammatory cytokines such as tumor necrosis factor (TNF)-α, interleukin (IL)-1, IL-6, galectin 3, soluble TNF receptor 1, and soluble TNF receptor 2 can be detected both in heart failure with reduced ejection fraction (HFrEF) and HFpEF. TNF provokes cardiomyocyte hypertrophy and impairs cardiac contractile function. TNF also induces dilation of the left ventricle as a result of extracellular matrix degradation. Several studies have also suggested the role of IL-6 in the progression of heart failure including its interaction with the vasculature and neurohormonal system to promote cardiac HF^31^.

**Figure 2:**
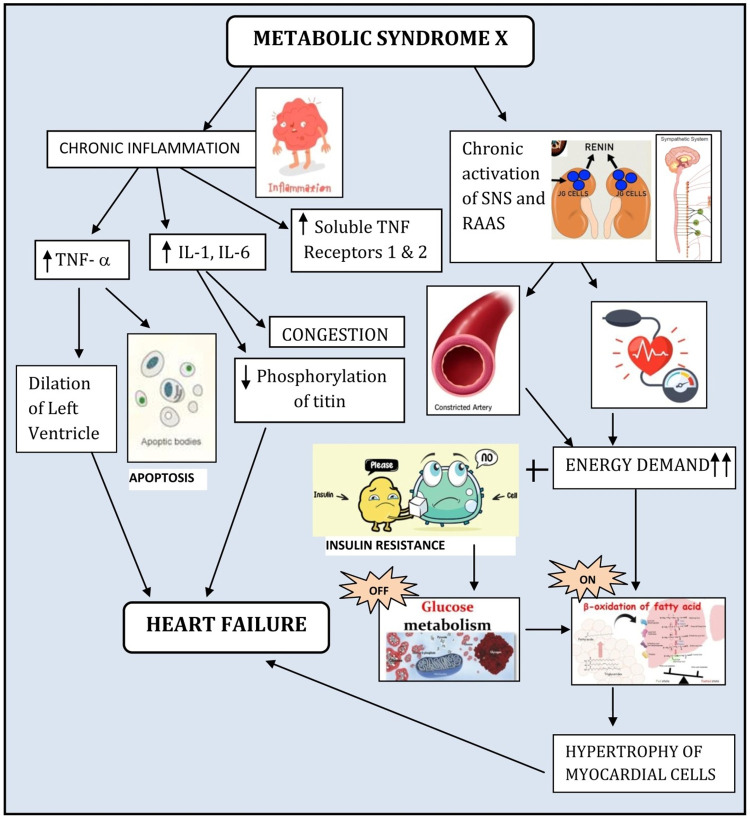
The mechanisms of heart failure in metabolic syndrome X patients

Overnutrition increases the levels of circulating free fatty acids and triglycerides, resulting in an increased accumulation of lipids and fatty acids in the myocardial cells by increased uptake. Activation of kinases results in increased serine phosphorylation of Insulin receptor substrate 1 (IRS-1)^32^. Insulin resistance occurs as a response to multiple stimuli leading to activation of s6 kinase. The response induced myocardial hypertrophy, cardiac fibrosis, impaired myocardial-endothelial signaling, and death of myocardial and endothelial cells. A study correlated a higher risk of heart failure incidence was related to a higher fasting insulin level. Prolonged Insulin resistance by the body can cause an increase in left atrial size, left ventricular mass, and alterations in transmitral velocity^33^. Hyperinsulinemia resulting from insulin resistance, along with an increase in adipokine levels and alterations of baroreceptor heart rate reflex are some key factors that give rise to chronic activation of the sympathetic autonomic nervous system. This leads to peripheral vasoconstriction and tachycardia. Although the metabolic demand of the heart increases in sympathetic stimulation, the metabolic supply to the tissues is decreased due to the transition from glycolysis pathway to fatty acid oxidation. The development of hypertrophy of cardiac myocytes results as a compensatory mechanism and failure to address the condition can thereby proceed to heart failure^31^ (Figure 2).

There is a paradoxical relationship between obesity and heart failure, especially in those with BMI 30-35. The mechanism of this still remains unclear, however, the increased lean mass that obese individuals have could play a critical role in improving long-term outcomes. Excess lean mass allows a higher cardiorespiratory fitness, which in turn improves heart failure prognosis; furthermore, the exact mechanism is unknown^34^.

## 6. Diagnosis

The diagnosis of heart failure begins with a thorough assessment of the patient's history and physical. Classic signs and symptoms of heart failure, such as dyspnea, dyspnea on exertion, orthopnea, peripheral edema, pathologic S3 and S4 heart sounds, crackles/rales at the lung bases, and jugular venous distention raise clinical suspicion of heart failure. It is of note that the history and physical examination provide low specificity and sensitivity to the diagnosis of heart failure^35^.

Electrocardiography (ECG), Chest X-ray, and Laboratory evaluation are initial diagnostic evaluations for patients who are suspected to have heart failure. ECG is particularly useful in patients that have HFrEF. These patients have significant ECG abnormalities, and the ECG provides high sensitivity and limited specificity for identifying HFrEF in these patients^36^. HFpEF on the other hand, displays a normal 12 lead ECG, though the presence of atrial fibrillation or paced rhythm increases the probability that HFpEF is present. A normal ECG makes left ventricle systolic dysfunction unlikely with a 98% negative predictive value^37^.

Chest radiographs are useful especially for patients presenting with dyspnea because they can provide information that helps differentiate heart failure from a primary pulmonary disease^38-40^. Cardiomegaly, Kerley-B lines, pleural effusion, and cephalization of the pulmonary vessels are findings suggestive of heart failure. Redistribution and cardiomegaly are the best predictors of increased preload and reduced ejection fraction, but neither is sufficient to definitively diagnose heart failure. alveolar edema, interstitial edema, and cephalization all have high specificity for HFrEF, but only cardiomegaly had a sensitivity of over 50 percent^39^. Chest X-rays are more limited in providing diagnostic value for patients with HFpEF where the sensitivity of cardiomegaly is 24 percent and pleural effusion is only 9 percent^41^.

It is recommended that the clinician obtain a complete blood count, urinalysis, serum electrolytes, blood urea nitrogen, serum creatinine, glucose, fasting lipid panel, liver function test, iron studies, assays for BNP or NT-proBNP, and thyroid-stimulating hormone levels for patients who are suspected to have heart failure. These laboratory studies provide essential information with regard to patients’ comorbidities, treatment approach, and potential causes or confounders of heart failure. In the context of the correct clinical presentation, BNP or NT-proBNP levels provide immense value to the diagnosis of heart failure, but should not be used in isolation to diagnose or exclude heart failure^42^. BNP or NT-proBNP levels have sensitivities of 93 percent and a specificity of 74 and 65 percent respectively for the diagnosis of heart failure^36^.

Once the clinician develops high suspicion of heart failure, echocardiography becomes key to the diagnosis. Left ventricular ejection fraction (LVEF), a measure of heart function collected by echocardiography, is used to diagnose and categorize heart failure into either HFpEF or HFrEF. If the LVEF is less than 50 percent, the patient is diagnosed with HFrEF, and if the LVEF is over or equal to 50 percent then the patient is diagnosed with HFpEF. While echocardiography is essential to the diagnosis of heart failure, it alone does not establish or exclude heart failure. Thus, a conclusive diagnosis cannot be made without considering the signs, symptoms, clinical features, laboratory values, chest radiography, and ECG of the patient being evaluated^35^ (Table 1).

**Table 1:** Overall accuracy of the clinical features of heart failure Clinical features with high sensitivity can help us rule out the disease when a patient has a negative result. Whereas clinical features with high specificity are useful when ruling in a patient's disease^37,43^.

Clinical features	Sensitivity (%)	Specificity (%)
History of MI	26	89
Dyspnea	87	51
Orthopnea	44	89
Peripheral edema	53	72
Lung crepatation	51	81
Elevated jugular venous pressure/ jugular venous distention	52	70
Cardiomegaly	27	85
Pathologic S3 or S4 heart sounds	11	99
Hepatomegaly	17	97
ECG in HFrEF	89	56
BNP	93	74
NT-proBNP	93	65
Chest radiograph	68	83
Echocardiography in HFpEF	85	96
Echocardiography in HFrEF	96	80

Metabolic syndrome X is diagnosed when the patient has central obesity with two or more of the following; raised triglycerides or specific treatment for this lipid abnormality, reduced HDL cholesterol or specific treatment for this lipid abnormality, raised blood pressure or treatment of previously diagnosed hypertension, and raised fasting plasma glucose or previously diagnosed type 2 diabetes. Metabolic Syndrome X is the most dangerous heart attack risk factor as patients with metabolic syndrome X are three times as likely to have a heart attack or stroke compared to people without the syndrome. It is imperative to diagnose and identify individuals with metabolic syndrome early, so that lifestyle interventions and treatment may prevent the development of diabetes mellitus and/or cardiovascular disease^44^.

## 7. Heart failure’s treatment in metabolic syndrome X

The treatment for heart failure in metabolic syndrome X is multifactorial and mainly targets the different components of metabolic syndrome X: obesity, hyperglycemia, hypertension, and dyslipidemia. These components can be treated using lifestyle modifications or pharmaceutical agents.

According to current guidelines, in patients with metabolic syndrome X and heart failure, the blood pressure should be less than 130/80 mmHg, cholesterol should be less than 100 mg/dL, and glycosylated hemoglobin levels should be less than 7 percent^45^. Lifestyle modifications like weight loss, exercise, or dietary changes can be beneficial in managing both heart failure and metabolic syndrome X. For example, aerobic exercise has been shown to have anti-hypertensive effects since it decreases systolic and diastolic blood pressure. Furthermore, it also decreases fasting blood glucose, triglycerides, and low-density protein concentrations. On the other hand, combined exercise has been shown to decrease diastolic blood pressure with minor improvements in high-density lipoprotein levels^43^. Moreover, a low carbohydrate, low fat, or Mediterranean diet helps improve the different components of metabolic syndrome X^46^. Yet, it is important to note that all of the above treatment options not only have a favorable effect on weight loss, but also on central obesity. It is essential to mention that even though obesity paradox exists, where obese and overweight patients with heart failure have a better prognosis than average weighted people with heart failure, there is little known about it. Therefore, the most acceptable and widely used therapy protocol still remains weight loss.

The pharmaceutical approach, much like lifestyle modifications, also targets the individual components of metabolic syndrome X. For hyperglycemia, the drug of choice is metformin. It is known to be a cardioprotective drug that is associated with better survival in patients with heart failure and diabetes mellitus. Moreover, it is safe to use in patients with diabetes mellitus and HFrEF, whereas insulin had a negative impact^47^. For patients with insulin resistance, medications like thiazolidinediones (TZDs), alpha-glucosidase inhibitors, and sulfonylureas have been effective. Interestingly, both metformin and TZDs have been shown to reduce levels of inflammatory markers like PAI-I and hs-CRP^48^. This is important because according to some studies, there is a possible correlation between inflammatory cytokines, heart failure, and metabolic syndrome X^18^. Therefore, both of these medications may prove to be beneficial in this regard too.

Angiotensin-converting enzyme inhibitors (ACE-I) and angiotensin receptor blockers (ARBs) are the mainstay of therapy for hypertension because they are cardioprotective drugs that can be used to treat patients with HFrEF. However, it is important to recognize that inotropic calcium channel blockers are contraindicated in this patient population. Furthermore, B-blockers, hydralazine, diuretics, and amlodipine can be used to treat systolic HF^31^.

For the treatment of dyslipidemia, the medication of choice is statins. Statins, like atorvastatin and simvastatin, have been associated with a reduction in HF-related hospitalizations in patients with pre-existing heart failure. But it is important to recognize that rosuvastatin, also a statin drug, has not been associated with decreasing cardiovascular events^45^. Hence to effectively treat heart failure in metabolic syndrome X, it is crucial to target the underlying components of metabolic syndrome using either lifestyle changes or medications.

## 8. Prognosis

Clinicians have long known that there is a direct correlation between patients with metabolic X and an increased risk of cardiovascular morbidity. The counterintuitive association between obesity and a declining prevalence of heart failure still raises questions. The paradox that overweight and obese people with established heart failure had a better short- and medium-term prognosis than leaner patients was well-documented in many epidemiologic studies in large heart failure cohorts. This association and the degree of cardiorespiratory fitness were related^7^.

Although sternal infections, supraventricular arrhythmias, and bleedings are more common, moderate obesity has not been demonstrated to be associated with perioperative morbidity and death^49^. Research has revealed that BMI (Body Mass Index) is ineffective at differentiating between metabolically healthy and unhealthy obesity and hence cannot be extrapolated to quantify the risk of cardiovascular morbidity^50^.

Although there is no doubt that weight loss enhances heart structure and function and lessens heart failure symptoms, no significant research has been done on how weight loss affects clinical events in heart failure. As a result, there are no recommendations for individuals with heart failure regarding the ideal body composition^51^.

Metabolically healthy obesity (MHO) and the "fat but fit" phenomena are terms used to describe patients who have been given an obese diagnosis based on BMI but who also have higher Cardiorespiratory fitness (CRF) and few metabolic abnormalities. Finally, we suggest that obese patients who present with an excess of body fat but no metabolic abnormalities should still be considered a population that is "at risk" and as such, they should be advised on how to change their lifestyle to improve their CRF and stop the onset of other cardiovascular disease risk factors like diabetes mellitus and impaired fasting glucose. This is known as primary prevention^52^.

## 9. Conclusion

The connection between heart failure and metabolic syndrome X is succinctly summarized in this study, with a focus on the pathophysiology, risk factors, diagnosis, therapy, and prognosis of the illness. It is well established that metabolic syndrome X is associated with an increased risk of cardiovascular morbidity and mortality. Therefore, the treatment of metabolic syndrome X remains to be a challenge in patients with heart failure. We have also mentioned the “obesity paradox” which explains that obesity can be a positive prognostic factor. Since it is not still well understood, the Obesity Paradox provides a gray area for future researchers as it can dramatically alter the course of treatment for heart failure in association with metabolic syndrome X.
